# Letter to the Editor Regarding “Effects of Intensity, Duration and Muscle Mass on Neuromuscular Fatigue and Blood Pressure After Isometric Handgrip Exercise”

**DOI:** 10.1002/hsr2.73076

**Published:** 2026-08-19

**Authors:** Maira Gul, Saira Rahimoon, Meva Ram, Ravi Das, Mohammad Mobin Kazemi

**Affiliations:** ^1^ Muhammad Medical College Mirpurkhas Sindh Pakistan; ^2^ Shaheed Mohtarma Benazir Bhutto Medical College Karachi Sindh Pakistan; ^3^ Jinnah Sindh Medical University Karachi Sindh Pakistan; ^4^ Ibn Sena Emergency Hospital Kabul Afghanistan

To the editor,

We read the article by Wang et al. with a lot of interest. The article is called ‘Effects of Intensity, Duration and Muscle Mass on Neuromuscular Fatigue and Blood Pressure After Isometric Handgrip Exercise’. It was published in Health Science Reports in 2026 [1]. We think the authors did a good job looking into how different isometric handgrip exercise protocols affect us. They also gave us some information about how neuromuscular fatigue and cardiovascular responses are related.

However, there are a few things that we think need to be discussed further. First, the study did not have a control group. This makes it hard to know if the changes they observed were really because of the isometric handgrip exercise. For example, Rodrigues et al. conducted a 12‐week study that included a control group. This made their findings more reliable when examining the effects of isometric handgrip training on blood pressure [2]. Therefore, future studies should include control groups. This will strengthen the validity of the findings and help us better understand the true effects of the intervention.

Second, Wang et al. only looked at the immediate effects after isometric handgrip exercise. They did not investigate whether these effects lasted over time. Oliveira et al. reviewed multiple studies and found that repeated isometric handgrip training, rather than a single session, helped reduce blood pressure in individuals with hypertension [3]. Wright et al. also found that 4 weeks of resistance training helped lower blood pressure in normotensive adults [4]. Therefore, future studies should evaluate the long‐term effects of isometric handgrip training to determine whether it provides sustained benefits.

Third, all participants in the study were healthy young adults. This may limit the applicability of the findings to populations that would benefit most from blood pressure reduction. Previous evidence has shown that isometric exercise training can reduce blood pressure in individuals with hypertension [5]. Therefore, future studies should include older adults and individuals with hypertension. This would help determine whether the findings are generalizable to broader and more clinically relevant populations.

In conclusion, the study by Wang et al. provides valuable information about isometric handgrip exercise protocols. However, several limitations may affect the applicability of the findings. These include the absence of a control group, the focus on only acute post‐exercise effects, and the inclusion of only healthy young adults. Addressing these limitations in future studies will improve our understanding of the role of isometric handgrip exercise in blood pressure management.

## Author Contributions

**Maira Gul:** conceptualization, writing – original draft, writing – review and editing. **Saira Rahimoon:** project administration, writing – review and editing, writing – original draft. **Meva Ram:** project administration, supervision, validation. **Ravi Das:** writing – review and editing, writing – original draft. **Mohammad Mobin Kazemi:** data curation, visualization, writing – original draft.

## Funding

The authors have nothing to report.

## Policy on Using ChatGPT and Similar AI Tools

This manuscript utilizes AI tools to enhance the wording. No part of this manuscript is entirely generated by AI.

## Data Availability

Data sharing not applicable to this article as no datasets were generated or analysed during the current study.
